# Functional state modelling approach validation for yeast and bacteria cultivations

**DOI:** 10.1080/13102818.2014.934550

**Published:** 2014-10-21

**Authors:** Olympia Roeva, Tania Pencheva

**Affiliations:** ^a^ Bioinformatics and Mathematical Modelling, Institute of Biophysics and Biomedical Engineering, Bulgarian Academy of Sciences, Sofia, Bulgaria; ^b^ QSAR and Molecular Modelling, Institute of Biophysics and Biomedical Engineering, Bulgarian Academy of Sciences, Sofia, Bulgaria

**Keywords:** functional state, modelling, *E. coli*, *S. cerevisiae*, cultivation

## Abstract

In this paper, the functional state modelling approach is validated for modelling of the cultivation of two different microorganisms: yeast (*Saccharomyces cerevisiae*) and bacteria (*Escherichia coli*). Based on the available experimental data for these fed-batch cultivation processes, three different functional states are distinguished, namely primary product synthesis state, mixed oxidative state and secondary product synthesis state. Parameter identification procedures for different local models are performed using genetic algorithms. The simulation results show high degree of adequacy of the models describing these functional states for both *S. cerevisiae* and *E. coli* cultivations. Thus, the local models are validated for the cultivation of both microorganisms. This fact is a strong structure model verification of the functional state modelling theory not only for a set of yeast cultivations, but also for bacteria cultivation. As such, the obtained results demonstrate the efficiency and efficacy of the functional state modelling approach.

## Introduction

Biotechnological processes, and especially cultivation processes, have enjoyed enormous advances in recent years. Due to their multidisciplinary nature, cultivation processes have attracted the interest of microbiologists, molecular biologists, bio- and chemical engineering, food and pharmaceutical chemists, etc. These complex processes are characterized with properties like non-linearity and non-stationarity. Thus, the development of accurate mathematical models essential for the design, optimization and high-quality control is still a challenging task.

When modelling such processes, the common approach is to develop a global non-linear model valid over the entire operation range. The main disadvantages of the global model are its very complex structure, inability to reflect possible metabolic changes that might occur during the process, as well as the non-stationarity of the parameters. To overcome these global model disadvantages, an alternative approach based on a multiple-model framework could be considered. The multiple-model approach allows some real phenomena or events to be reflected, leading to process description with simpler local models; and offers possibilities for direct incorporation of high-level and qualitative plant knowledge into the model.[1]

Considering the applications of the multiple-model approach for biotechnological processes, the one considered more convenient for further process control is the functional state modelling (FSM) approach. The FSM approach was originally developed by Zhang et al. [2,3] for aerobic yeast growth processes and several works have already shown its benefits.[4,5] Its applicability to the mathematical modelling of *Saccharomyces cerevisiae* CEN.PK haploid batch and *S. cerevisiae* DY 7221 fed-batch cultivations has been shown.[6,7] This approach can be used not only for process predictions, but also for early stabilization of process,[7] robust control,[8] model-based control [9] and design of multiple-model non-linear adaptive control algorithms.[10] This provoked us to attempt to validate the theory of the FSM approach not only for yeast cultivations, but also for mathematical modelling of bacteria cultivations.

Although the concept of FSM was originally developed for yeast cultivation processes,[2,3] it could be applied for the modelling of *Escherichia coli* cultivations as well.[11] Based on [12] it is demonstrated that there is an analogy between the *S. cerevisiae* and *E. coli* growth curves.[5,11]

Yeasts are one of the most important microorganisms with various applications in food and bread production, beer and wine fermentation, etc. In addition, yeasts include some of the most widely used model organisms in genetic engineering and cell biology due to the fact that their metabolic pathways are well known. That is why yeast cultivations are often used as a test process for new methods or ideas.

Another common model microorganism is *E. coli*, whose cultivation is, in many cases, the only economically viable way to produce many biopharmaceuticals.[5] Since 1997, when the entire *E. coli* genome was published, scientific research on this bacterium has accelerated.[13]

The aim of this paper is to present a validation of the FSM approach for the cultivation of two different types of microorganisms, yeast (*S. cerevisiae*) and bacteria (*E. coli*). Experimental data from three different real cultivations are used. For identification of the model parameters, genetic algorithms (GA) are applied.[14] This metaheuristic technique has already proved effective in solving complex, non-linear optimization tasks.[5,15–18]

## Functional state modelling approach

The common modelling approach when such complex processes are modelled is to develop a non-linear model that describes the process dynamics over the entire operating range. As a result, one global process model is constructed. Unfortunately, this approach has a lot of disadvantages. The main one is the difficulty in finding a suitable model structure and, further, the invariably complex structure. Another disadvantage is that the global models do not consider the parameter non-stationarity. Due to metabolic changes it is logical that the parameter values will not be constant during the entire process. There are some cases when the modelling studies are performed to identify simple, easy-to-use and robust models, suitable to support the engineering tasks of process optimization and control. Often, however, similar model simplification leads to misunderstanding of process characteristics. In order to avoid these disadvantages, an alternative approach for model development based on multiple-model framework is considered. Using this approach, complicated problems are decomposed into subproblems that can be solved independently. Then, the individual solutions of the decomposed problems lead to the global solution of the complex problem.

The multiple-model-based approach is an appropriate tool for monitoring and control of complex processes such as bioprocesses.[1–4,6,7,19] When applying such an approach, the process is decomposed into different operating stages. Thus, based on relatively simpler models more accurate description of the process dynamics is achieved.[4] This methodology allows for partial transparency of process knowledge to the modelling procedure.

The main idea of the FSM approach is to consider the division of the process into macrostates termed functional states (FSs) according to specific behaviour.[5] During each FS, a certain metabolic pathway dominates over the process behaviour. Zhang et al. [2,3] have proposed a division of the whole yeast growth process into five FSs, considering the batch and/or fed-batch modes.

Table 1 illustrates the interrelationships of the different FSs during fed-batch yeast cultivation.[2,3] FS IV, which is characteristic only of batch cultures, is omitted because a fed-batch mode is considered here. The detailed description of all FSs according to certain metabolic pathways can be found in [2,3].

**Table 1.** Functional states interrelationships.

Functional state	Rule
*First functional state* (*FS I*) first product synthesis state	*S* > *S*_crit_ and O_2_ > O_2crit_
*Second functional state* (*FS II*) mixed oxidative state	*S* ≤ *S*_crit_ and O_2_ ≥ O_2crit_ and *P* > 0
*Third functional state* (*FS III*) complete sugar oxidative state	*S* ≤ *S*_crit_ and O_2_ ≥ O_2crit_ and *P* = 0
*Fifth functional state* (*FS V*) second product synthesis state	*S* ≤ *S*_crit_ and O_2_ < O_2crit_ and *P* > 0

Note: *S* is the concentration of substrate (g·L^−1^); O_2_ is the dissolved oxygen concentration (%); *P* is the concentration of the product (g·L^−1^), which is ethanol in the case of *S. cerevisiae* cultivation and acetate in the case of *E. coli* cultivation.

The rules presented in Table 1 can be used for recognition of FS during fed-batch cultivation of *E. coli* as well, based on the proved analogies between the fermentation metabolisms of *S. cerevisiae* and *E. coli*.[11] According to the mass balance and following [2,3], the mathematical description of the considered fed-batch cultivation for all FSs can be presented as(1) 
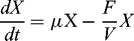

(2) 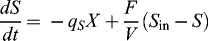

(3) 
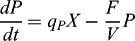

(4) 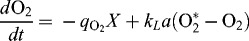

(5) 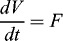
where *X* is the biomass concentration (g·L^−1^); *F* is the influent flow rate (h^−1^); *V* is the bioreactor volume (L); *S*_in_ is the influent glucose concentration (g·L^−1^); O_2_* is the dissolved oxygen saturation concentration (%); μ is the specific growth rate (see Table 2) (h^−1^); *q_S_*, *q_P_* and 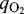 are specific rates (see Table 2) (g·g^−1^·h^−1^); and *k_L_a* are volumetric transfer coefficients (h^−1^).

**Table 2.** Local model parameter functions.

Specific rates	FS I	FS II	FS III	FS V
μ				
*q_S_*		**	**	
*q_P_*			0	
				

Note: *S*_crit_ is the critical level of substrate concentration (g·L^−1^); O_2crit_ is the critical level of dissolved oxygen concentration (%); μ_max_ is the maximum specific growth rate (h^−1^); *Y_i_*_/*j*_ is the yield coefficient (g·g^−1^); *k_j_* is the saturation constant (g·L^−1^ or %).

Each FS of the process is described by the so-called local model, which is valid only in this FS. The structures of μ, *q_S_*, *q_P_* and 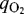 vary depending on the FS (Table 2).

## Materials and methods

### Cultivation conditions

The experimental data for the fed-batch cultivations of *S. cerevisiae* and *E. coli* were obtained in the Institute of Technical Chemistry, University of Hannover (Germany). The conditions for both cultivations are presented in Table 3. The biomass and products (ethanol or acetate) were measured offline, while the substrate (glucose) and dissolved oxygen were measured online.

**Table 3.** Conditions of fed-batch cultivations.

Cultivation conditions	S. cerevisiae*	*E. coli*
Volume (L)	1.5	2.0
Temperature (°C)	30	35
pH	5.7	6.9
Stirrer speed (r·min^−1^)	500	900–1800
*S*_in_ (g·L^−1^)	50	100
Set point (g·L^−1^)	0.08/0.05*	0.1

*Two different runs of *S. cerevisiae* fed-batch cultivation were carried out: the first one at set point 0.08 and the second one at set point 0.05 g·L^−1^ glucose.

### Model parameter identification

Several authors have already shown the benefits, efficiency and efficacy of GA for more accurate and adequate parameter identification of the cultivation process models.[20–24] That is why GA were used for identification procedures of the local models presented in Table 2. The detailed descriptions of the GA operators and parameters tuning can be found in [15]. For completeness, here we present the GA operators and parameters that were applied in the parameter identification procedures in this study: encoding (binary); crossover (double point); mutation (bit inversion); selection (roulette wheel selection); fitness function (linear ranking); and generation gap (0.97); crossover rate (0.70); mutation rate (0.1); precision of binary representation (20); number of individuals (100); number of generations (100).

A minimization of a distance measure between real values of state variables and predicted ones was used as an optimization criterion:(6) 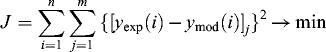
where *J* is the optimization criterion; *n* is the number of measurements for each state variable; *m* is the number of state variables; *y*_exp_ is the experimental data vector; and *y*_mod_ is the model predicted data vector. In the considered case of fed-batch cultivations, the criterion *J* was formed for the data vector *y* = [*S*, *X*, *P*, O_2_].

genetic algorithm Toolbox [25] in MATLAB environment was used for parameter identification procedures for all local models considered here, for both yeast and bacteria cultivations.

## Results and discussion

### Simulation verification of the FSM approach on yeast and bacteria

Based on Zhang et al. [2,3] and the experiments with the *S. cerevisiae* strain and cultivations, the following critical values for *S* and O_2_ concentrations were assumed for the fed-batch mode considered here: (1) first cultivation run:

*S*_crit_ = 0.08 g·L^−1^ and O_2crit_ = 18%;
  and (2) second cultivation run:
*S*_crit_ = 0.05 g·L^−1^ and O_2crit_ = 18%.

Taking into account the rules for FS recognition shown in Table 1, for the first cultivation run of *S. cerevisiae* the first product (i.e., ethanol) synthesis state (FS I) was identified, since dissolved oxygen and glucose concentrations are above the corresponding critical levels and at the same time there is ethanol production. In this state, pyruvate is accumulated and ethanol is produced.

For the second cultivation run of *S. cerevisiae*, the FS II (mixed oxidative state) was identified for the data set. The yeast growth process enters this state when the sugar concentration in the broth decreases below the critical level. In this state, both sugar and ethanol are co-metabolized to produce energy and the intermediates for yeast growth.

Based on the experiments with the *E. coli* strain and cultivations, the following values for substrate and dissolved oxygen critical levels were assumed:


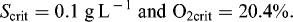


In the case of the *E. coli* fed-batch cultivation process, three FSs are identified: (1) first product (i.e., acetate) synthesis state (FS I) – from 6.7 h (beginning of the cultivation) to 7.2 h of the cultivation, when the dissolved oxygen and the glucose concentrations are above the corresponding critical levels; (2) mixed oxidative state (FS II) – from 7.2 to 10.5 h cultivation time, when the sugar concentration decreases below the critical level and there is sufficient dissolved oxygen in the broth; and (3) second product (i.e., acetate) synthesis state (FS V) – from 10.5 to 11.6 h cultivation time, when both glucose and dissolved oxygen concentrations are below the corresponding critical levels.

Parameter identification procedures were performed for each of the recognized FSs with corresponding local models in both yeast and bacteria cultivations. The values of estimated parameters applying GA are listed in Table 4.

**Table 4.** Estimated values of local model parameters.

	FS I		FS II		FS V
Parameters	*S. cerevisiae*	*E. coli*	*S. cerevisiae*	*E. coli*	*E. coli*
μ*_S_* (h^−1^)	0.24	0.45	0.95	0.58	0.55
μ*_E_* (h^−1^)	–	–	0.34	0.32	–
*k_S_* (g·L^−1^)	0.28	0.03	0.11	0.04	0.093
*k_E_* (g·L^−1^)	–	–	5.01	5.44	–
*Y_S_*_/*X*_ (g·g^−1^)	0.09	0.46	0.45	0.49	0.33
*Y_P_*_/*S*_ (g·g^−1^)	1.48	0.045	0.67	0.22	5.26
*k_L_a* (h^−1^)	80	52.49	98.27	87.27	30.54
(g·g^−1^)	0.001	0.096	–	–	0.063
(g·g^−1^)	–	–	0.001	14.58	–
(g·g^−1^)	–	–	0.0013	26.09	–
(g·L^−1^)	–	–	–	–	0.028

**Figure 1.**
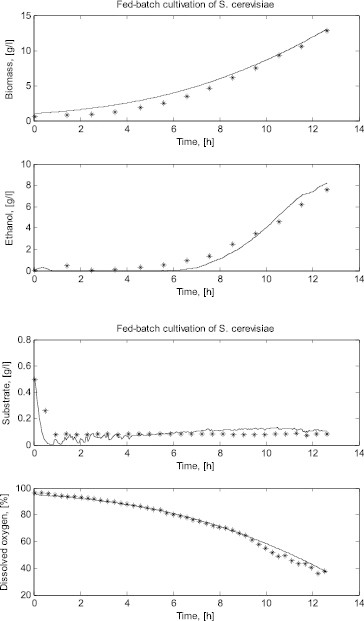
Comparison between a simulated and a real fed-batch *S. cerevisiae* cultivation process (run I).

Both the real fed-batch cultivation of *S. cerevisiae* (I run) trajectories for the biomass and ethanol concentrations and the simulated ones are presented in Figure 1, as well as the variation of substrate and dissolved oxygen concentrations.

**Figure 2.**
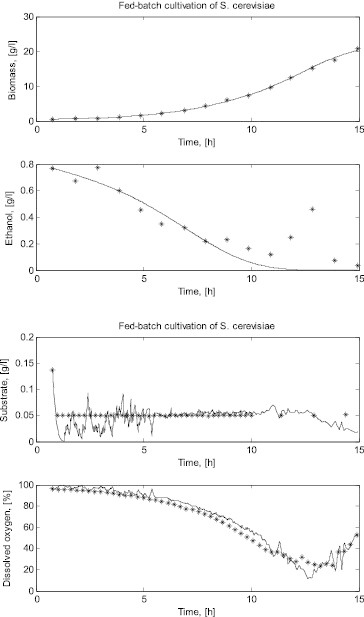
Comparison between a simulated and a real fed-batch *S. cerevisiae* cultivation process (run II).

The real and the simulated trajectories for the II run fed-batch cultivation of *S. cerevisiae* are shown in Figure 2.

**Figure 3.**
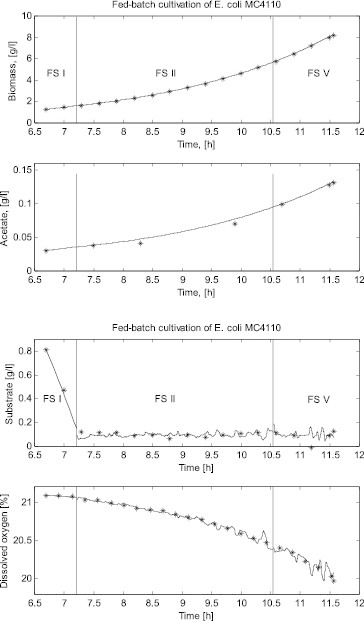
Comparison between a simulated and a real fed-batch *E. coli* cultivation process.

Figure 3 presents the measured cultivation trajectories and the simulated ones for all the FSs in the *E. coli* fed-batch cultivation process. In this presentation of the results, the initial values for the simulation in the new FS are the last simulated values in the previous FS so that the trajectories are continuous.

The simulation results showed that the original local models presented by Zhang et al. [2,3] successfully predict the variation of glucose consumption, biomass concentration, product (ethanol or acetate) formation and dissolved oxygen consumption during the two fed-batch cultivation processes considered here. Especially the last more complex cultivation – with a domination of different metabolic pathways during one cultivation process – proves the need of using the FSM approach. The high degree of accuracy of the simulation results demonstrates the validity of the structure of local models for both yeast and bacteria cultivations. The results also indicate that the FSM approach can help to better understand the behaviour of a process and simplify the process modelling.

## Conclusions

In the present investigation, the FSM approach was validated for modelling of yeast and bacteria cultivation processes. Based on experimental data for *S. cerevisiae* fed-batch cultivation and the rules for FS recognition, only one FS was identified during the two different runs of the cultivation: first product synthesis state (for the first cultivation run) and mixed oxidative state (for the second cultivation run). The same two FSs were identified in the *E. coli* fed-batch cultivation process, as well as an additional one: second product (i.e., acetate) synthesis state. The simulation results showed very good prediction of both the *S. cerevisiae* and *E. coli* process behaviour and indicate that the two processes can be adequately modelled with the FSM approach with good efficiency.

## References

[cit0001] Murray-Smith R, Johansen TA (1997). Multiple model approaches to modelling and control.

[cit0002] Zhang X-Ch, Visala A, Halme A, Linko P (1994). Functional state modeling and fuzzy control of fed-batch aerobic baker's yeast process. J Biotech..

[cit0003] Zhang X-Ch, Visala A, Halme A, Linko P (1994). Functional state modelling approach for bioprocesses: local models for aerobic yeast growth processes. J Process Control..

[cit0004] Pencheva T, Vassileva S, Ilkova T, Georgieva Y, Hitzmann B, Tzonkov S (2004). Multimodel approach for modelling of biotechnological processes. Biotechnol Biotechnol Equipment..

[cit0005] Roeva O, Pencheva T, Georgieva Y, Hitzmann B, Tzonkov S (2004). Implementation of functional state approach for modelling of *Escherichia coli* fed-batch cultivation. Biotechnol Biotechnol Equipment..

[cit0006] Vilums S, Grigs O, Mednis M Application of functional state modelling approach for yeast *Saccharomyces cerevisiae* batch fermentation state estimation.

[cit0007] Vilums S, Kozlinskis E, Brusbardis V Application of functional state modelling approach for yeast *Sacharomyces cerevisiae* fed-batch fermentation modelling.

[cit0008] Renard F, Wouwer A, Valentinotti S, Dumur D (2006). A practical robust control scheme for yeast fed-batch cultures − an experimental validation. J Process Control..

[cit0009] Nagy Z (2007). Model based control of a yeast fermentation bioreactor using optimally designed artificial neural networks. Chem Eng J..

[cit0010] Slavov Ts, Roeva O (2014). Multiple non-linear model adaptive control of cultivation process: hardware-in-the-loop simulation of control system. CR Acad Bulg Sci..

[cit0011] Roeva O, Pencheva T, Viesturs U, Tzonkov S (2006). Modelling of fermentation processes based on state decomposition. Int J Bioautomation.

[cit0012] Nielsen J, Villadsen J (1994). Bioreaction engineering principles.

[cit0013] Keseler IM, Collado-Vides J, Santos-Zavaleta A, Peralta-Gil M, Gama-Castro S, Muniz-Rascado L, Bonavides-Martinez C, Paley S, Krummenacker M, Altman T, Kaipa P, Spaulding A, Pacheco J, Latendresse M, Fulcher K, Sarker M, Shearer AG, Mackie A, Paulsen I, Gunsalus RP, Karp PD (2011). EcoCyc: a comprehensive database of *Escherichia coli* biology. Nucleic Acids Res.

[cit0014] Goldberg DE (2006). Genetic algorithms in search, optimization and machine learning.

[cit0015] Angelova M, Pencheva T (2011). Tuning genetic algorithm parameters to improve convergence time. Int J Chem Eng..

[cit0016] Angelova M, Pencheva T (2012). Algorithms improving convergence time in parameter identification of fed-batch cultivation. CR Acad Bulg Sci..

[cit0017] Fidanova S, Roeva O, Ganzha M, Fidanova S (2013). ACO and GA for parameter settings of *E. coli* fed-batch cultivation model. Recent advances in computational optimization, Studies of computational intelligence.

[cit0018] Roeva O (2012). Real-world application of genetic algorithms.

[cit0019] Hjersted J, Henson MA (2005). Population modeling for ethanol productivity optimization in fed-batch yeast fermenters.

[cit0020] Angelova M, Atanassov K, Pencheva T (2012). Purposeful model parameters genesis in simple genetic algorithms. Comput Math Appl..

[cit0021] Chen LZ, Chen XD, Nguang SK, Chen LZ, Nguang SK, Chen XD (2006). Optimization of fed-batch fermentation processes using genetic algorithms based on cascade dynamic neural network models. Modelling and optimization of biotechnological processes, Studies in computational intelligence.

[cit0022] Chong Y, Yan A, Yang X, Cai Y, Chen J (2012). An optimum fermentation model established by genetic algorithm for biotransformation from crude polydatin to resveratrol. Appl Biochem Biotechnol..

[cit0023] Pencheva T, Angelova M, Atanassov K, Vasant P (2014). Genetic algorithms quality assessment implementing intuitionistic fuzzy logic. Handbook of research on novel soft computing intelligent algorithms: theory and practical applications.

[cit0024] Roeva O., Sabelfeld KK, Dimov I (2013). Chapter 21. A comparison of simulated annealing and genetic algorithm approaches for cultivation model identification. Monte Carlo methods and applications.

[cit0025] Chipperfield AJ, Fleming PJ, Pohlheim H, Fonseca CM (1994). Genetic algorithm toolbox for use with MATLAB. User's guide. Version 1.2.

